# Evaluation of gene expression change in eosinophilic gastroenteritis 

**Published:** 2019

**Authors:** Mohammad-Mahdi Zadeh-Esmaeel, Mostafa Rezaei-Tavirani, Nayeb Ali Ahmadi, Reza Vafae

**Affiliations:** 1 *Skin Research Center, Shahid Beheshti University of Medical Sciences, Tehran, Iran *; 2 *Proteomics Research Center, Faculty of Paramedical Sciences, Shahid Beheshti University of Medical Sciences, Tehran, Iran *; 3 *Proteomics Research Center, Shahid Beheshti University of Medical Sciences, Tehran, Iran*

**Keywords:** Eosinophilic gastroenteritis, gene, protein-protein interaction network

## Abstract

**Aim:**

Screening differentially expressed genes (DEGs) related to Eosinophilic gastroenteritis (EG) to introduce possible biomarkers.

**Background:**

EG as a rare gastrointestinal disorder is characterized with gastrointestinal bleeding, crampy generalized abdominal pain, diarrhea, nausea, vomiting, and weight loss. In this study gene expression profile of patients is analysis via protein-protein interaction (PPI) analysis to reveal new prospective of disease.

**Methods:**

Top significant genes of gene expression profiles of 5 gastric antrum EG patients and 5gastric antrum control from GEO which were matched via boxplot analysis were screened via PPI network by using Cytoscape software and STRING database. Numbers of 20 top nodes of query DEGs based on degree value were introduced as central nodes which 7 critical central genes among them were identified. Gene ontology enrichment for the 20 central genes was done by using CluGO. Action map for the central genes was performed by applying CluePedia.

**Results:**

Among 20 central nodes, TXN, PRDX2, NR3C1, GRB2, PIK3C3, AP2B1 and REPS1 were recognized as critical central genes. Nine biological terms were determined that most of them were involved in the transport processes.

**Conclusion:**

The introduced possible biomarkers can be used in the differential diagnosis of the disease and also in treatment of disorder.

## Introduction

 Eosinophilic gastroenteritis (EG) is classified as a rare gastrointestinal disorder emerging with heterogeneous profile of physical manifestations such as gastrointestinal bleeding, crampy generalized abdominal pain, diarrhea, nausea, vomiting, and weight loss and/or various combinations of these symptoms. Clinical presentations simply vary and are related to the layer affected by abnormal eosinophilic infiltration (1). Based on the depth of involvement, EG can be categorized as mucosal, muscular, and serosal types (2). Although the disease, first described in 1937, can be observed in any part of the gastrointestinal tract, the stomach and small intestine are the most involved regions (3). In the United States, a prevalence of ranging from 8.4 – 28 per 100,000 cases, with a slightly elevated incidence over the last 50 years, has been described (4). Even though environment factors comprising higher socioeconomic status, parasitic infestation, and food diet may be risk factors, a notable contribution has been suggested by genetic factor (4, 5). Of note, some assessments have revealed the association between EG and other autoimmune disorders such as systemic lupus erythematosus, ulcerative colitis, and celiac disease (6-8).

Despite the fact a clear pathogenesis and etiology is not yet established, the role of components of inflammation as well as hypersensitivity reaction pathways may play a role. Interestingly, in one study it was demonstrated that 50% of EG patients had a positive history of allergy including rhinitis, asthma, drug allergy, and eczema (9). Literature, moreover, suggest the role of enhanced serum immunoglobulin E (IgE) and peripheral blood eosinophilia in the pathophysiology of this condition. Immunohistochemical investigations in diseased intestinal wall also highlight that cytokines such as interleukin (IL)-3, IL-5, granulocyte macrophage colony stimulating factor, and delayed TH2 cell-mediated allergic mechanisms are also considered to exert essential roles in the expansion, recruitment, and activation of eosinophils to the gastrointestinal (GI) tract, which is the pathogenic mechanism underlying the EG hallmark (2). Likewise, chemokines, called eotaxin 1 and α4b7 integrin, are thought to participate in eosinophilic homing inside the intestinal wall. In addition, other elements, including IL-4, IL-13, tumor necrosis factor (TNF)-alpha, and leukotrienes, have been named to increase the eosinophilic trafficking as well as prolonging the eosinophilic activity (10-12).

To date, no comprehensive risk factor or etiologic studies have been performed for EG (13). All the above data collectively suggest an immune dysregulation for the pathogenesis of EG with a significant, yet not entirely well-examined, potential role of genetic factors. Given the fact that imaging has revealed an inappropriate role in supporting the EG diagnosis, as well as the very little interest in invasive methods such as endoscopy (14), a revolutionized classification of biomarkers and related dysregulated molecular mechanisms is strongly required. 

## Methods

Gene expression profile of 5 gastric antrum EG patients and 5gastric antrum control patients were extracted from GEO. Data are presented as GSE54043 entitle “Global gene expression profile of gastric antrum tissue of patients with eosinophilic gastritis” in GEO. RNA samples of patients were extracted from gastric biopsy of 5 normal patients and 5 EG individuals. Gene expression distribution of profiles was matched via boxplot analysis. The top 250 significant (P-value≤0.001) DEGs were determined. Cutoff FC≥1.5 was considered and the uncharacterized DEGs were excluded. The screened DEGs included constructing PPI network.

The network was constructed by Cytoscape v 3.6.0 (15) and STRING as its plugin. Due to weak interactions between DEGs in the network, numbers of 100 relevant neighbors were added to the query DEGs. The network was analyzed by Network analyzer as an application of Cytoscape. Numbers of 20 top nodes of query DEGs based on degree value were introduced as central nodes. Degree distribution of nodes was performed to determine the scale free type of the network. Gene ontology enrichment for the 20 central genes was done by using CluGO (16) and the related biological terms were clustered in the significant groups. Action map for the central genes was performed by applying CluePedia (17). 

## Results

**Figure 1:**
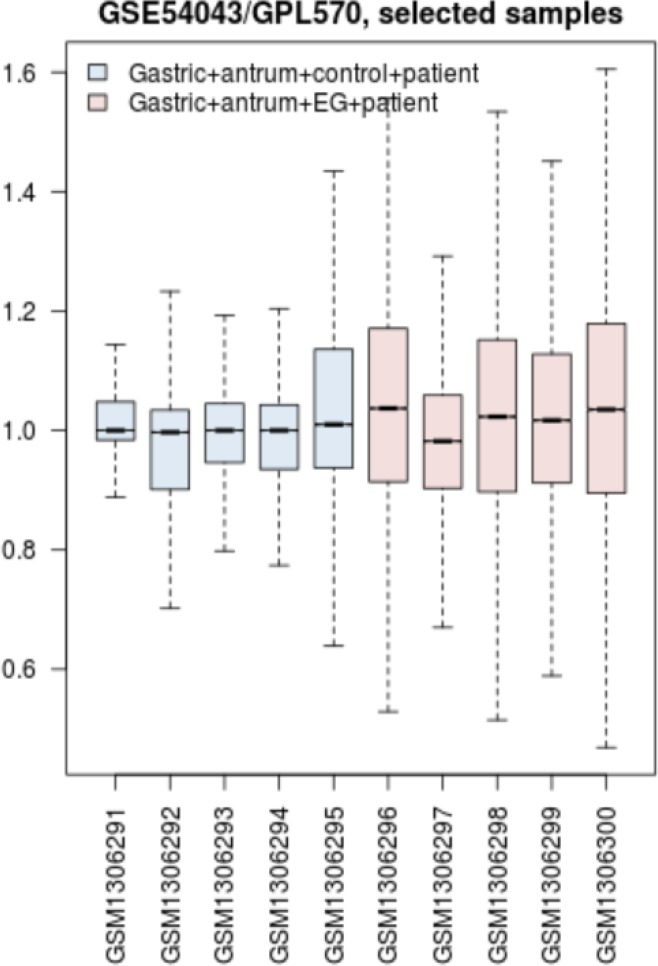
Boxplot analysis of samples is illustrated. The sample codes are presented in the horizontal axis and the normalized amounts of expression is shown in the vertical axis

Statistical analysis is required to validate comparison between samples. Boxplot analysis of samples is shown in the figure 1.

**Table 1:** 20 central genes which play role in eosinophilic gastritis are shown. Description is provided by Cytoscape software and is abstracted. D, BC and CC refer to degree, betweenness centrality and closeness centrality respectively

Gene Name	Description	D	BC	CC	Stress
TXN	Surface-associated sulphydryl protein; Participates in various redox reactions. Contributes to the response to intracellular nitric oxide (by inhibition caspase-3 activity). Induces the FOS/JUN AP-1 DNA-binding activity.	40	0.008	0.503	4620
NR3C1	Nuclear receptor subfamily 3, group C, member 1 (glucocorticoid recetor); Isoform Alpha-D3: Has lowest transcriptional activation activity of all isoforms created by alternative initiation. Has transcriptional repression activity; Nuclear hormone receptors	38	0.017	0.500	7468
GRB2	Growth factor receptor-bound protein 2; Adapter protein that provides a critical link between cell surface growth factor receptors and the Ras signaling pathway; SH2 domain containing	37	0.006	0.490	3324
PRDX2	Thioredoxin-dependent peroxide reductase 1; Plays a role in cell protection against oxidative stress. Might participate in the signaling cascades of growth factors and tumor necrosis factor-alpha.	34	0.006	0.488	3530
PIK3C3	Phosphatidylinositol 3-kinase, catalytic subunit type 3; Plays a role in multiple membrane trafficking pathways.	28	0.002	0.474	1402
AP2B1	Clathrin assembly protein complex 2 beta large chain; Component of the adaptor protein complex 2 (AP-2). Adaptor protein complexes function in protein transport via transport vesicles in different membrane traffic pathways.	27	0	0.453	168
REPS1	RalBP1-associated Eps domain-containing protein 1; May coordinate the cellular actions of activated EGF receptors and Ral-GTPases; EF-hand domain containing	26	0	0.450	84
PPIG	Peptidylprolyl isomerase G (cyclophilin G); PPIases accelerate the folding of proteins.	22	0.003	0.466	1498
TIA1	TIA1 cytotoxic granule-associated RNA binding protein; Possesses nucleolytic activity against cytotoxic lymphocyte target cells. May be involved in apoptosis.	21	0.003	0.444	1288
UBE2L3	Ubiquitin-conjugating enzyme E2L 3; UBE2 is involved in progression through the cell cycle. Regulates nuclear hormone receptors transcriptional activity. May play a role in myelopoiesis.	21	0.002	0.462	976
FUBP1	Far upstream element (FUSE) binding protein 1; Regulates MYC expression.	20	0.002	0.434	1188
HNRNPDL	Heterogeneous nuclear ribonucleoprotein D-like; Acts as a transcriptional regulator.	20	0.004	0.429	1638
FBP1	D-fructose-1,6-bisphosphate 1-phosphohydrolase 1; Plays a role in regulating glucose sensing and insulin secretion of pancreatic beta-cells. Appears to modulate glycerol gluconeogenesis in liver. Important regulator of appetite and adiposity.	19	0.016	0.437	4516
NMUR2	G-protein coupled receptor TGR-1; Receptor for the neuromedin-U and neuromedin-S neuropeptides.	19	0.001	0.406	414
MAP2	Microtubule-associated protein 2; The exact function of MAP2 is unknown but MAPs may stabilize the microtubules against depolymerization.	18	0	0.423	230
PPWD1	Peptidylprolyl isomerase domain and WD repeat-containing protein 1; Putative peptidylprolyl isomerase (PPIase). PPIases accelerate the folding of proteins.	17	0	0.413	0
PTAFR	Platelet-activating factor receptor; Receptor for platelet activating factor, a chemotactic phospholipid mediator that possesses potent inflammatory, smooth- muscle contractile and hypotensive activity.	17	0	0.415	406
RBBP6	P53-associated cellular protein of testis; May play a role as a scaffold protein to promote the assembly of the p53/TP53-MDM2 complex, resulting in increase of MDM2-mediated ubiquitination and degradation of p53/TP53.	16	0	0.436	454
PFKP	ATP-dependent 6-phosphofructokinase, platelet type; Catalyzes the phosphorylation of D-fructose 6-phosphate to fructose 1,6-bisphosphate by ATP, the first committing step of glycolysis.	15	0.001	0.430	594
HACE1	HECT domain and ankyrin repeat containing E3 ubiquitin protein ligase 1; Acts as a regulator of Golgi membrane dynamics during the cell cycle.it may playing a role in host defense against pathogens.	14	0	0.406	6

**Table 2:** LogFC is represented for the 7 top central nodes based on degree value (the nodes of first phase in figure 4). The red highlighted DEGs are up-regulated and the rest are down-regulated

**Gene Name**	**D**	**BC**	**CC**	**Stress**	**LogFC**
TXN	40	0.008	0.503	4620	0.713
NR3C1	38	0.017	0.500	7468	-1.052
GRB2	37	0.006	0.490	3324	0.657
PRDX2	34	0.006	0.488	3530	0.608
PIK3C3	28	0.002	0.474	1402	-0.668
AP2B1	27	0	0.453	168	0.614
REPS1	26	0	0.450	84	-0.626

**Figure 2:**
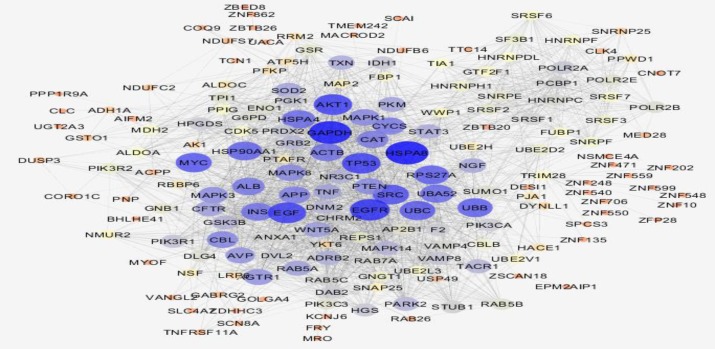
Main connected component of PPI network of gastric antrum EG patients in comparison with control is presented. The nodes are layout based on degree value; bigger size refers to higher value of degree. Red to blue color refers to higher value of degree

**Figure 3:**
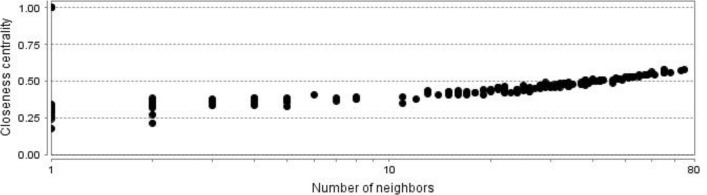
Closeness centrality distribution for PPI network of gastric antrum EG patients in comparison with control is shown

**Figure 4:**
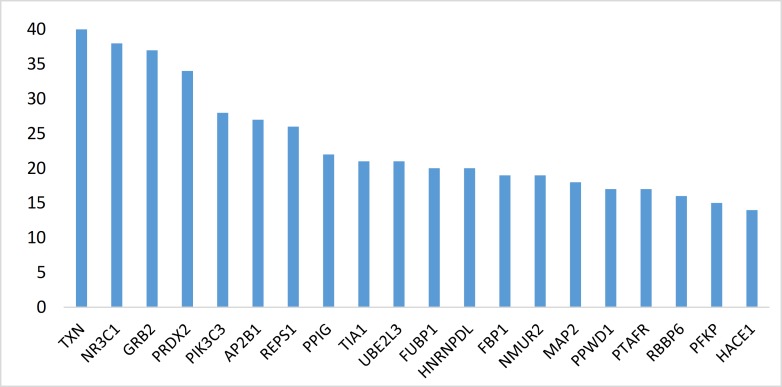
Degree value of 20 central nodes is shown

**Figure 5:**
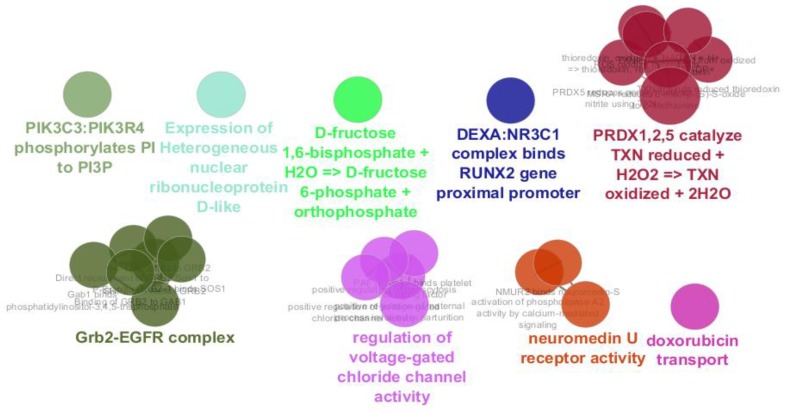
Gene ontology finding related to the 20 central genes of PPI network of gastric antrum EG patients in comparison with control is presented. Biological terms are clustered in the 9 groups. kappa score = 0.4 was considered

**Figure 6:**
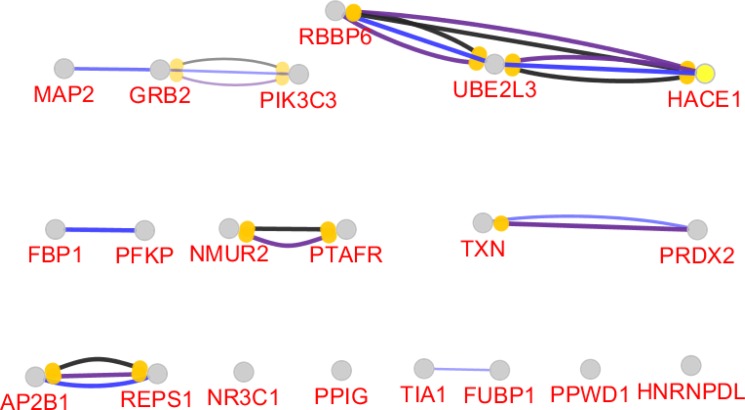
Action map related to the 20 central genes of PPI network of gastric antrum EG patients in comparison with control is shown. Blue, black and purple colors refer to binding, catalyze and reaction actions respectively. kappa score = 0.4 was considered

The data are comparable due to median center distribution of data. In this figure 5 gene expression profiles of gastric antrum control patients and 5 gastric antrum EG patients are compared as samples. Among top 250 significant DEGs (P-value≤0.001), numbers of 154 characterized DEGs were identified with FC≥1.5 which were included to construct PPI network. The numbers of 109 DEG among 154 characterized DEGs were recognized by STRING database. Since there was weak relationship between the 109 query DEGs in PPI network, numbers of 100 neighbors were added to the 109 ones to construct the network. The network including 24 isolated genes, two paired components and a main connected component was constructed. The main connected component which will call network contains 181 nodes and 2141 edges. Among 109 query genes, 81 DEGs were included in the network. The network is shown in the figure 2. 

The network is a scale free network. In this type of network there are few central nodes which are differentiated from the other nodes by higher numbers of links or the other values of centrality parameters. In figure 3 scale free type of network is shown. As it is depicted in the figure 2, most of hub-nodes belong to the neighbor nodes and few query genes are characterized as hub. For better screening of the query genes, 20 top of them based on degree value were selected as central genes (see table 1). For better understanding and possible screening of central nodes, degree values of central genes are shown in the figure 4. As it is depicted in the figure 4 degree value change is a biphasic curve including the 7 first ones and the other 13 nodes. LogFC for the 7 nodes of the first phase is represented in the table 2. Gene ontology finding related to the 20 central genes is presented in the figure 5. The 27 biological terms are clustered in nine groups. Since action map is a suitable tool to show relationship between genes, in figure 6 action types between the 20 central nodes are represented.

## Discussion

There are many studies about EG epidemiology and etiology which explain its mechanism and relationship to the other gastric disorders (18, 19). Since effective treatment with minimal side effects and also protection of diseases requires molecular knowledge especially genetics aspects of disorders, here prominent genes which play crucial role in EG are introduced and discussed. As it is depicted in the figure 1 gene expression distribution in all samples are median center; therefore, the samples are comparable. In this figure also it is appeared that gene expression distribution for patients has wide range relative to the normal ones which refers to the differences between patients and normal samples in overall. Based on figures 2 and 3 the constructed network is scale free so there are limited DEGs that can be separated from the others and play critical role in the network. Arbitrary 20 top query DEGs were selected as central DEGs; however, some of them may be more important relative to the other ones. As it is shown in the table 1 the central nodes have the higher values of the other centrality parameters except betweenness parameter. Most of hub-nodes which are characterized with lower value of betweenness are ranked in the bottom of table 1. The hub-nodes that are characterized with higher value of betweenness are called hub-bottlenecks (20). The hub-bottleneck nodes are ranked in the up part of table 1. For better resolution, the critical central nodes including 7 DEGs were identified via figure 4. So, the roles of 20 central DEGs in EG are investigated via gene ontology (see figures 5 and 6) and prominent roles of 7 critical central DEGs are discussed in more details. The following terms which are presented in the table 1 are affected in EG via deregulation of 20 central genes:

Redox reactions such as the response to intracellular nitric oxide, transcriptional repression activity, nuclear hormone receptors, critical link between cell surface growth factor receptors and the Ras signaling pathway, cell protection against oxidative stress, signaling cascades of growth factors and tumor necrosis factor-alpha, multiple membrane trafficking pathways, protein transport via transport vesicles in different membrane traffic pathways, cellular actions of activated EGF receptors and Ral-GTPases, proteins folding acceleration, apoptosis, cell cycle, myelopoiesis, regulation of MYC expression, transcriptional regulation, regulation of glucose sensing and insulin secretion of pancreatic beta-cells, modulation of glycerol gluconeogenesis in liver, regulation of appetite and adiposity, receptors of some neuropeptides, stabilization of the microtubules against depolymerization, inflammation, smooth- muscle contractile and hypotensive activity, assembly of the p53/TP53-MDM2 complex, the first committing step of glycolysis, host defense against pathogens, FOS/JUN AP-1 DNA-binding activity. MYC, FOS, TP53, JUN, and EGFR are highlighted as related genes to the query DEGs. There are evidence that dysregulation of these related genes is correlated to cancer (21). It can be concluded that EG can be considered as risk factor of gastric cancer.

As it is shown in the table 2, TXN and PRDX2 are two critical central DEGs that are up-regulated in EG. Closed relationship between both TXN and PRDX2 is appeared in the action map (see figure 6). The largest GO group in figure 2 is PRDX1, 2, 5 catalyze TXN reduced + H2O2 => TXN oxidized + 2H2O. In this reaction Peroxiredoxin 1 (PRDX1), PRDX2, and PRDX5 in the cytosol reduce hydrogen peroxide (H2O2) with thioredoxin yielding oxidized thioredoxin and water (22, 23).

The second critical central element in the table 1 and 2 is glucocorticoid receptor (NR3C1) that is down-regulated in EG. It is reported that dexamethasone (DEXA) activates NR3C1in mice. The activated NR3C1 is able to bind glucocorticoid receptor response element in RUNX2 gene (24). Investigation indicates that RUNX2 has a possible oncogenic role in esophageal carcinoma. PI3K/ AKT and ERK pathways are two pathways that are activated by RUNX2 (25).

GRB2 and PIK3C3 are the other two critical central DEGs which are connected in the action map in figure 6. GRB2-EGFR complex which is highlighted as an important group in the figure 5 corresponds to the effect of GRB2 on internalization of signaling via EGFR that leads to macropinocytic pathway (26). As it is described in the REACTOME pathway database (https://reactome.org/content/detail/R-BTA-6798174), PIK3C3 is involved in the cytosolic compartment of phagocytic vesicles that catalyze Pi to Pi3P via conversion of ATP into ADP. This product (Pi3P) is necessary to catalyze NADPH into NADP+. Relationship between GRB2-PIK3C3 refers to importance of membrane trafficking control in EG. Perhaps using sodium cromoglycate (a stabilizer of mast cell membranes) as drug in treatment of EG patients confirms this relationship (27-29).

 AP2B1-REPS1 relationship in figure 6 and their opposite expression change indicate that there is negative correlation between the two rest critical central genes. FCs of AP2B1 and REPS1 are 0.614 and -0.626. Again protein transport via transport vesicles in different membrane traffic pathways is highlighted for AP2B1 in the table 1 which reflects importance of membrane instability in EG. Doxorubicin transport and regulation of voltage-gated chloride channel activity are the two important transporter groups which are presented in the figure 5. It is reported that activated RalA and RalBP1/RLIP76 promote endocytosis which leads to regulation of several biological processes. Oncogenesis, cell migration, transcription, apoptosis, proliferation and differentiation are the known processes that are affected by REPS1 (30, 31). These evidence indicate that the introduced 7 critical central genes can be considered as EG biomarkers and cell membrane is the critical cellular compartment in the EG.

In conclusion TXN, PRDX2, NR3C1, GRB2, PIK3C3, AP2B1 and REPS1 can be introduced as potential biomarker for EG. It is suggested that more details of finding be investigated via additional research in the field.
